# Effects of paliperidone extended release on the symptoms and functioning of schizophrenia

**DOI:** 10.1186/1472-6904-12-1

**Published:** 2012-01-06

**Authors:** Min-Wei Huang, Tsung-Tsair Yang, Po-Ren Ten, Po-Wen Su, Bo-Jian Wu, Chin-Hong Chan, Tsuo-Hung Lan, I-Chao Liu, Wei-Cheh Chiu, Chun-Ying Li, Kuo-Sheng Cheng, Yu-Chi Yeh

**Affiliations:** 1 Institute of Biomedical Engineering, National Cheng Kung University, Tainan 70403, Taiwan; 2 Department of Psychiatry, Chiayi Branch, Taichung Veterans General Hospital, Chia-Yi 60090, Taiwan; 3 Department of Psychiatry, Cardinal Tien Ken-Sin Hospital, Taipei 23148, Taiwan; 4 Department of Psychiatry, Show Chwan Memorial Hospital, Changhua 50008, Taiwan; 5 Department of Psychiatry, Chu-Tung Branch, National Taiwan University Hospital, Hsinchu 31064, Taiwan; 6 Department of Psychiatry, Yuli Hospital, Hualien 98147, Taiwan; 7 Department of Psychiatry, Taichung Veterans General Hospital, Taichung 40705, Taiwan; 8 Department of Psychiatry, Cathay General Hospital, Taipei 10630, Taiwan; 9 Medical Devices Innovation Center, National Cheng Kung University, Tainan 70403, Taiwan

## Abstract

**Background:**

We aimed to explore relations between symptomatic remission and functionality evaluation in schizophrenia patients treated with paliperidone extended-release (ER), as seen in a normal day-to-day practice, using flexible dosing regimens of paliperidone ER. We explored symptomatic remission rate in patients treated with flexibly dosed paliperidone ER by 8 items of Positive and Negative Syndrome Scale (PANSS) and change of Personal and Social Performance (PSP) scale.

**Method:**

This was a 12-week multicenter, open-label, prospective clinical study conducted in in-patient and out-patient populations. Flexible dosing in the range 3-12 mg/day was used throughout the study. All subjects attended clinic visits on weeks 0, 4, 8, and 12 as usual clinical practice for the 12-week observation period. Data were summarized with respect to demographic and baseline characteristics, efficacy measurement with PANSS scale, PSP, and social functioning score, and safety observations. Descriptive statistics were performed to identify the retention rate at each visit as well as the symptomatic remission rate. Summary statistics of average doses the subjects received were based on all subjects participating in the study.

**Results:**

A total of 480 patients were enrolled. Among them, 426 patients (88.8%) had evaluation at week 4 and 350 (72.9%) completed the 12-week evaluation. Patients with at least moderate severity of schizophrenia were evaluated as "mild" or better on PANSS scale by all 8 items after 12 weeks of treatment with paliperidone ER. There was significant improvement in patients' functionality as measured by PSP improvement and score changes. Concerning the other efficacy parameters, PANSS total scale, PSP total scale, and social functioning total scale at the end of study all indicated statistically significant improvement by comparison with baseline. The safety profile also demonstrated that paliperidone ER was well-tolerated without clinically significant changes after treatment administration.

**Conclusions:**

Although the short-term nature of this study may limit the potential for assessing improvements in function, it is noteworthy that in the present short-term study significant improvements in patient personal and social functioning with paliperidone ER treatment were observed, as assessed by PSP scale.

**Trial Registration:**

Clinical Trials. PAL-TWN-MA3

## Background

Schizophrenia is a severe form of mental illness affecting about 24 million people worldwide (7 per 1000 adult population), mostly in the age group 15-35 years. Although the incidence is low (3/10,000), the prevalence is high due to chronicity [1]. Deficits in social functioning can be observed throughout the course of schizophrenia: in the early stages, during acute exacerbations, and over long-term maintenance treatment.

The early course of schizophrenia typically includes a prodromal phase characterized by nonspecific symptoms and behaviors, a formal onset/deteriorative stage with active psychosis, cognitive impairment, negative symptoms, and social deficits, and a period of several years following the initial episode that often includes repeated episodes of psychosis with a progressive increase in residual symptoms and functional decline. There is general agreement that approximately 5 years after the initial psychotic episode patients enter a chronic, but relatively more stable phase with no marked further decline in functioning or increase in residual symptoms [1-5].

With the advancement of new medications, treatment goals of patients with schizophrenia were raised. On the one hand, remission, instead of response was recognized as the optimal treatment goal for patients with schizophrenia. Research on treatments for schizophrenia focused predominantly on symptom improvement; however, outcomes such as cognition, health-related quality of life, and social functioning are now being recognized as important indices of treatment success. The Remission in Schizophrenia Working Group (RSWG) chose 8 items of PANSS (delusions, unusual thought content, hallucinatory behavior, conceptual disorganization, mannerisms/posturing, blunted affect, social withdrawal, and lack of spontaneity) as determinants for the definition of remission [6]. Several studies have already implemented this concept and found that patients achieving remission status had better performance in neuropsychological tests and greater social and occupational functions [7-9]. On the other hand, functionality became an important focus of treatment in psychotic patients. Patients who returned to normal life were considered as undergoing "truly recovery" [5]. The Personal and Social Performance (PSP) scale, developed to measure patients' personal and social functionality, is a convenient tool in clinical practice. Several clinical trials measured patients' functioning as study endpoints with this scale [10].

Typically with antipsychotic drugs, dose titration to the maintenance dose is recommended. Paliperidone is available in a formulation using extended-release (ER) osmotic release technology (OROS^®^), hereinafter referred to as paliperidone ER. This formulation was designed to deliver paliperidone at a controlled rate over a 24-hour period, resulting in a gradual increase in plasma concentration after the first intake and low peak-to-trough fluctuation at steady state [11]. Paliperidone is the major metabolite of risperidone. It is a prolonged release oral atypical antipsychotic for the treatment of schizophrenia. Based on preclinical experiments and clinical investigations, paliperidone is an effective and safe antipsychotic medication for the treatment of schizophrenia. Some studies showed that paliperidone ER significantly improved symptoms and functioning in schizophrenia patients regardless of time since diagnosis [4,12-15]. The phase III well-controlled pivotal efficacy and safety studies were performed using randomly applied fixed dosages (3, 6, 9, 12, or 15 mg/day) of paliperidone ER. In daily clinical practice, however, flexible dosing is applied based on the individual needs of patients. The pivotal studies were also performed in well-defined homogenous groups of subjects with schizophrenia. In daily clinical practice, however, a more diverse population is treated, e.g. having higher rates of comorbidities and/or comedications. Pivotal studies also used an initial washout period. Therefore, no data for direct transition from a variety of oral antipsychotics to flexibly dosed paliperidone ER are available today.

In most treatment-related clinical trials, response, measured with certain percent improvement of rating scales, is used as outcome determinant. However, approaches focusing on psychotic patients' real life functioning are the main interest of clinical practice. Achieving symptom-free and normal life ought to be the key measure in clinical studies. Moreover, understanding symptom-free function is of great value for treatment goals in clinical practice. Therefore, we designed this study to explore symptomatic remission and functionality evaluation in patients treated with paliperidone ER, as seen in normal day-to-day practice, using flexible dosing regimens.

## Methods

### 2.1 Study Design

This was a 12-week, multicenter, open-label, prospective clinical study conducted in inpatients and outpatients. Throughout the study flexible dosing in the range 3-12 mg/day was used so as to allow investigators to adjust the dosage of each subject based on individual needs. In general, the recommended paliperidone ER dose was 6 mg once daily, although some subjects benefited from lower or higher doses in the recommended dose range.

After obtaining informed consent, baseline characteristics, PANSS scale, PSP, and social functioning scale were assessed and recorded. Treatment of these subjects was decided by clinicians' opinion. As for patients with pharmacotherapy, dosing was flexible throughout the study period according to investigators' discretion based on individual subjects' clinical response to and tolerability of study drug. During the study observation period, subjects attended clinic visits on weeks 0, 4, 8, and 12 as usual clinical practice. At the preplanned clinic visits, PANSS and PSP scale, reports of adverse events, and treatment information were recorded. Subjects could withdraw from this study at any time; reasons of withdrawal or loss of follow-up were recorded. The study was approved by the Institutional Review Board of Cathay General Hospital (protocol no. PAL-TWN-MA3).

### 2.2 Patient Population

Participants were male or female and met DSM-IV diagnostic criteria above aged 18 years. They were drug naïve; their previous treatment was considered unsuccessful due to one or more of the following reasons: lack of efficacy, lack of tolerability or safety, lack of compliance, and/or other reasons. Subjects or their legally acceptable representatives had signed an informed consent document indicating that they understood the purpose of and procedures required for the study and were willing to participate in the study. Female subjects were postmenopausal for ≥ 1 year, surgically sterile, abstinent, or, if sexually active, agreed to practice an effective method of birth control before entry and throughout the study. Effective methods of birth control included prescription hormonal contraceptives, contraceptive injections, intrauterine devices, double-barrier method, contraceptive patch, and male partner sterilization. Female subjects also had a negative urine pregnancy test at screening.

Individuals were excluded from the study if the patients were on clozapine, paliperidone ER, any conventional depot neuroleptic or Risperdal^® ^Consta^® ^during the last 3 months. Subjects experienced serious unstable medical condition including known clinically relevant laboratory abnormalities, history of neuroleptic malignant syndrome, hypersensitivity to paliperidone ER or risperidone or inability to swallow the study medication whole with the aid of water (subjects may not chew, divide, dissolve, or crush the study medication because this may affect the release profile) were excluded. Pregnant or breast-feeding woman and participation in another investigational drug trial in the 30 days prior to selection were also excluded from the study. Of course, employees of the investigator or study center, persons with direct involvement in the proposed study or other studies under the direction of that investigator or study center, or family members of the employees or the investigator were not allowable.

At each visit subjects received the amount of medication required until the next visit. Subjects from any oral antipsychotic medication could be switched to an effective dose of paliperidone ER without the need for titration. Subjects could be cross-tapered in different ways from their previous antipsychotic medication, e.g. a decrease of the previous antipsychotic drug may occur at the time of or after initiation of paliperidone ER. The period of cross-tapering also varied among subjects, since both dosing and timing of transition depended on relevant individual subject characteristics such as kind and severity of current symptoms or adverse events, course of previous relapses and rehospitalizations, or type and dose of previous antipsychotic medication (e.g. with or without anticholinergic and/or sedating properties).

The neuropsychiatric symptoms of schizophrenia were assessed by 30-item PANSS scale, which provides a total score (sum of the scores of all 30 items). Each scale is rated 1 (absent) to 7 (extreme). The PANSS assessment was performed by a qualified rater defined as a trained clinician. If possible, for a given subject, the same rater assessed this scale at all visits. Subjects were interviewed at each visit to assess the psychiatric symptoms of schizophrenia.

The following 8 items were used as determinants for remission:

-P1 Delusions

-P2 Conceptual disorganization

-P3 Hallucinatory behavior

-G9 Unusual thought content

-G5 Mannerisms and posturing

-N1 Blunted affect

-N4 Social withdrawal

-N6 Lack of spontaneity/flow of conversation

Subjects were rated for their personal and social performance at each visit by PSP scale. This scale assessed the degree of difficulty a subject exhibited over a 1-month period within 4 domains of behavior: socially useful activities, personal and social relations, self-care, and disturbing and aggressive behavior. The score ranged from 1 to 100, divided into 10 equal intervals to rate the degree of difficulty (absent to very severe) in each of the 4 domains. Subjects with scores 71-100 had a mild degree of difficulty, 31-70 varying degrees of disability, and ≤ 30 functioning so poorly as to require intensive supervision.

### 2.3 Statistical Analysis

Data were analyzed on intent-to-treat (ITT) principle. All statistical tests were performed with an alpha level of 0.05. Descriptive analysis of the demographic variables and other baseline line variables was conducted using measures of central tendency and variation for quantitative variables and frequency distributions for categorical variables. Assessment of safety included computation of the incidence of AEs and of discontinuation due to AEs, and presented in a frequency distribution table.

Two cohorts were introduced into the study:

- All enrolled subjects (overall);

- An ITT population comprising all enrolled subjects who received paliperidone ER at least once and provided ≥ 1 post-baseline efficacy measurement.

The efficacy analysis was mainly performed on the ITT population, but also performed on all enrolled subjects. The safety profile was assessed for the ITT population.

Data were summarized with respect to demographic and baseline characteristics, efficacy measurement with PANSS scale, PSP, and social functioning score, and safety observations. Descriptive statistics were performed to identify the retention rate at each visit as well as the symptomatic remission rate. Summary statistics of average doses that subjects received were based on all subjects participating in the study. Descriptive analysis was performed including frequency and percentage for categorical parameters, and mean, standard deviation, minimum, and maximum for continuous parameters. Descriptive analyses comprised summary statistics and 95% confidence intervals (95%CI). The paired t test was also performed to compare changes in scores of continuous variables.

The assessment of safety was based mainly on the frequency of AEs. The Medical Dictionary for Regulatory Activities (MedDRA, Version 12.1) AE dictionary was used to map AEs to preferred terms and system organ class. Patients reporting an individual preferred term AE and the total number of patients reporting at least one adverse event per system organ class were tabulated. Each AE based on preferred terminology was counted only once for a given subject for each group. The frequency and percent AEs (preferred terms and system organ class) are presented. Descriptive statistics were provided to evaluate the changes of vital signs at each scheduled time-point.

## Results

**Figure 1:**
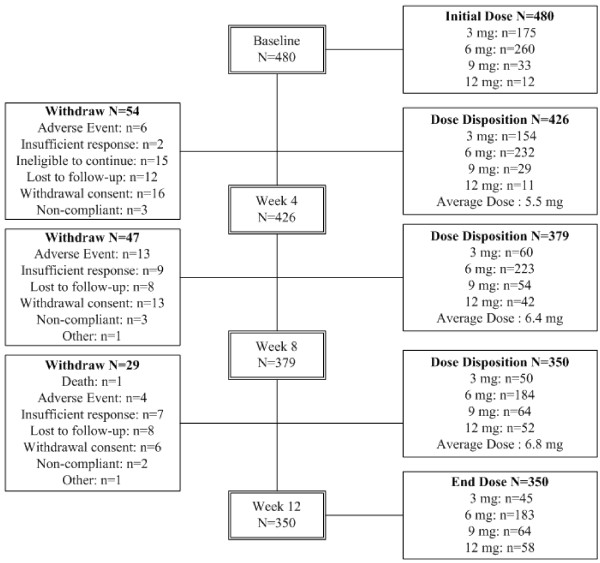
**Patient Disposition**. A total of 480 patients were enrolled. Among them, a total of 426 subjects (88.8%) had evaluation at week 4 and 350 (72.9%) completed the 12-week evaluation. The details of patient disposition are summarized.

A total of 480 patients were enrolled. Among them, a total of 426 subjects (88.8%) had evaluation at week 4 and 350 (72.9%) completed the 12-week evaluation. The details of patient disposition are summarized in Figure 1.

Reasons of patient withdrawal before week 4 were AEs (n = 6), insufficient response (n = 2), ineligible to continue (n = 15), lost to follow-up (n = 12), consent withdrawn (n = 16), and noncompliance (n = 3). Therefore these 54 patients did not have any safety and efficacy evaluation, resulting in 426 subjects included in the ITT population.

The initial dose disposition at baseline for these 426 patients was 3 mg/day for 154 patients, 6 mg/day for 232 patients, 9 mg/day for 29 patients, and 12 mg/day for 11 patients. The average dosage was 5.5 mg/day. The end doses for 350 subjects who completed the study were distributed as 45 patients with 3 mg/day, 183 patients with 6 mg/day, 64 patients with 9 mg/day, and 58 patients with 12 mg/day.

The withdrawal reasons are summarized in Table 1. Overall, 130 subjects (27.1%) discontinued the study prematurely. The details are as follows: 1 subject (0.2%) died, 12 subjects (4.8%) withdrew because of adverse events, 18 (3.8%) subjects withdrew because of insufficient response, 15 subjects (3.1%) were ineligible to continue, 28 subjects (5.8%) were lost to follow-up, 35 subjects (7.3%) withdrew their consent, 8 (1.7%) subjects discontinued because of non-compliance, and 2 subjects (0.4%) because of other reasons.

**Table 1:** Summary of Withdrawal Reason

Time	Overall (N = 480)	ITT (N = 426)
Total	130 (27.1%)	76 (17.8%)
Death	1 (0.2%)	1 (0.2%)
Adverse Event	23 (4.8%)	17 (4.0%)
Insufficient Response	18 (3.8%)	16 (3.8%)
Ineligible to Continue	15 (3.1%)	0 (0.0%)
Lost to Follow-up	28 (5.8%)	16 (3.8%)
Withdrew Consent	35 (7.3%)	19 (4.5%)
Non-Compliant	8 (1.7%)	5 (1.2%)
Other	2 (0.4%)	2 (0.5%)

Summary statistics of demographic characteristics for the overall and ITT populations are listed in Table 2. In the ITT population, there were more men (55.9%) than women (44.1%). The mean age was 40.4 (range, 17-72) years; median age was 40 years. Subjects' schizophrenia subtype distribution was paranoid 61.7%, undifferentiated 18.9%, disorganized 10.8%, residual 7.3%, catatonic 1.2%, and other subtypes 0.2%. Overall, 33.3% of the subjects had symptom onset > 10 years but < 20 years. There were 4.9% of subjects with history of drug abuse. The results of all enrolled subjects were similar to those of the ITT population.

**Table 2:** Summary of Demographics

Characteristics		Overall (N = 480)	ITT (N = 426)
Sex	Male	262 (54.6%)	238 (55.87%)
	Female	218 (45.4%)	188 (44.13%)

Age (years)	N	< 480 >	< 426 >
	Mean (SD)	40.3 (10.6)	40.4 (10.6)
	Median	39.5	40.0
	Min. ~ Max.	16.0 ~ 72.0	17.0 ~ 72.0

Diagnosis	Paranoid	300 (62.5%)	263 (61.74%)
	Disorganized	46 (9.6%)	46 (10.80%)
	Catatonic	6 (1.3%)	5 (1.17%)
	Undifferentiated	88 (18.3%)	80 (18.78%)
	Residual	37 (7.7%)	31 (7.28%)
	Other	3 (0.6%)	1 (0.23%)

In/Out Patient	In-patient	218 (45.4%)	207 (48.6%)
	Out-Patient	262 (54.6%)	219 (51.4%)

Symptom onset (years)	Unspecified	46 (9.6%)	41 (9.62%)
	< = 5	95 (19.8%)	78 (18.31%)
	> 5~ < = 10	83 (17.3%)	74 (17.37%)
	> 10~ < = 20	153 (31.9%)	142 (33.33%)
	> 20~ < = 30	78 (16.2%)	67 (15.73%)
	> 30	25 (5.2%)	41 (9.62%)

Drug abuse	No	437 (91.0%)	386 (90.61%)
	Yes	23 (4.8%)	21 (4.93%)
	Unspecified	20 (4.2%)	19 (4.46%)

**Figure 2:**
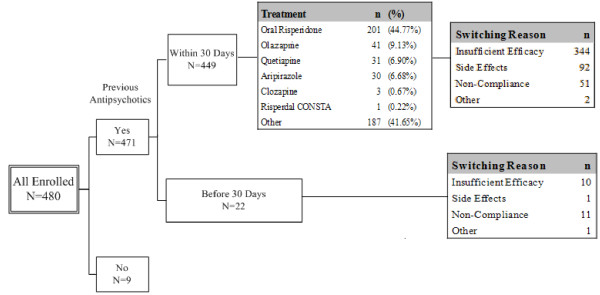
**Summary of switching reasons of previous antipsychotic treatment of all enrolled patients**. For the enrolled population, there were 9 subjects who did not receive any antipsychotics at enrollment. For the remaining 471 subjects, 449 received antipsychotics within 30 days prior to enrollment. The treatments included oral risperidone for 201 subjects (44.77%), olanzapine for 41 subjects (9.13%), quetiapine for 31 subjects (6.90%), aripiprazole for 30 subjects (6.68%), and other treatments for 187 subjects (41.65%). The major reason of switching treatment was insufficient efficacy, accounting for a total of 344 subjects. AEs (92 subjects), noncompliance (51 subjects), and other (2 subjects) were the reasons for switching. Twenty-two subjects received antipsychotics > 30 days prior to enrollment. The switching reasons were insufficient efficacy (n = 10), side effects (n = 1), noncompliance (n = 11), and other (n = 1).

**Figure 3:**
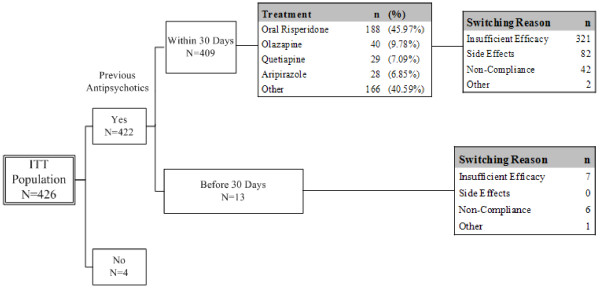
**Summary of switching reasons of previous antipsychotic treatment of ITT population**. For the ITT population, there were 4 subjects who did not receive any antipsychotics at enrollment. For the remaining 422 subjects, 409 received antipsychotics within 30 days prior to enrollment. The treatments included oral risperidone for 188 subjects (45.97%), olanzapine for 40 subjects (9.78%), quetiapine for 29 subjects (7.09%), aripiprazole for 28 subjects (6.85%), and other treatments for 166 subjects (40.59%). The major reason of switching treatment was insufficient efficacy, accounting for a total of 321 subjects. AEs (82 subjects), noncompliance (42 subjects), and other (2 subjects) were the reasons for switching. Thirteen subjects received antipsychotics > 30 days prior to enrollment. The switching reasons were insufficient efficacy (n = 7), noncompliance (n = 6), and other (n = 1).

The reasons for subjects switching their treatments are displayed for all enrolled patients and the ITT population in Figures 2 and 3, respectively. For the ITT population, there were 4 subjects who did not receive any antipsychotics at enrollment. For the remaining 422 subjects, 409 received antipsychotics within 30 days prior to enrollment. The treatments included oral risperidone for 188 subjects (45.97%), olanzapine for 40 subjects (9.78%), quetiapine for 29 subjects (7.09%), aripiprazole for 28 subjects (6.85%), and other treatments for 166 subjects (40.59%). The major reason of switching treatment was insufficient efficacy, accounting for a total of 321 subjects. AEs (82 subjects), noncompliance (42 subjects), and other (2 subjects) were the reasons for switching. Thirteen subjects received antipsychotics > 30 days prior to enrollment. The switching reasons were insufficient efficacy (n = 7), noncompliance (n = 6), and other (n = 1).

Table 3 summarizes previous antipsychotic treatment received for consecutive 3 months. The most frequently used antipsychotics were oral risperidone (207 subjects; 48.6%) for the ITT population. The results of all enrolled subjects were similar to those of the ITT population.

**Table 3:** Summary of Previous Antipsychotics Treatment Received for Consecutive 3 Months

Antipsychotic Treatments	Overall (N = 480)	ITT (N = 426)
Oral Risperidone	224 (46.67%)	207 (48.59%)
Olanzapine	52 (10.83%)	49 (11.50%)
Quetiapine	35 (7.29%)	32 (7.51%)
Aripiprazole	30 (6.25%)	28 (6.57%)
Clozapine	14 (2.92%)	10 (2.35%)
Risperdal CONSTA	3 (0.63%)	1 (0.23%)
Other	125 (26.04%)	110 (25.82%)

Table 4 summarizes the complicating diseases for subjects. For the ITT population, the most commonly complained complications were psychiatric (329 subjects; 77.23%), gastrointestinal (145 subjects; 34.04%), and neurological disorders (104 subjects; 24.41%), respectively.

**Table 4:** Summary of Concurrent Disease with Incidence ≧ 5%

System	Overall (N = 480)	ITT (N = 426)
Psychiatric	364 (75.83%)	329 (77.23%)
Gastrointestinal	156 (32.50%)	145 (34.04%)
Neurological	114 (23.75%)	104 (24.41%)
Cardiovascular	77 (16.04%)	74 (17.37%)
Endocrine	71 (14.79%)	62 (14.55%)
Respiratory	34 (7.08%)	31 (7.28%)
Ears, Nose, Throat	29 (6.04%)	28 (6.57%)
Musculoskeletal	32 (6.67%)	31 (7.28%)

Dose disposition of study medication paliperidone ER of the ITT population and completed population is presented in Table 5 and Table 6, respectively. In the ITT population, the number of subjects who started paliperidone ER treatment with the initial dose of 3 mg/day and increased to 6, 9, and 12 mg/day at the end of study was 69, 17, and 18, respectively. There were 43 and 30 subjects with the initial dose of 6 mg/day and increased to 9 and 12 mg/day, respectively, at the end of study, whereas 11 subjects with the initial dose of 9 mg/day increased to 12 mg/day at the end of study. All subjects with initial dose of 12 mg/day remained on 12 mg/day till the end of study. The completed population had a similar dose pattern of study dose disposition.

**Table 5:** Summary of Dose Disposition of ITT Population

ITT Population		Initial Dose			
		
		3 mg/day	6 mg/day	9 mg/day	12 mg/day
Dose at the End of Study	3 mg/day	50	7	0	0
	6 mg/day	69	152	0	0
	9 mg/day	17	43	18	0
	12 mg/day	18	30	11	11

**Table 6:** Summary of Dose Disposition of Complete Study Subjects

Complete Study Subjects		Initial Dose			
		
		3 mg/day	6 mg/day	9 mg/day	12 mg/day
Dose at the End of Study	3 mg/day	40	5	0	0
	6 mg/day	61	122	0	0
	9 mg/day	11	42	11	0
	12 mg/day	15	26	8	9

**Figure 4:**
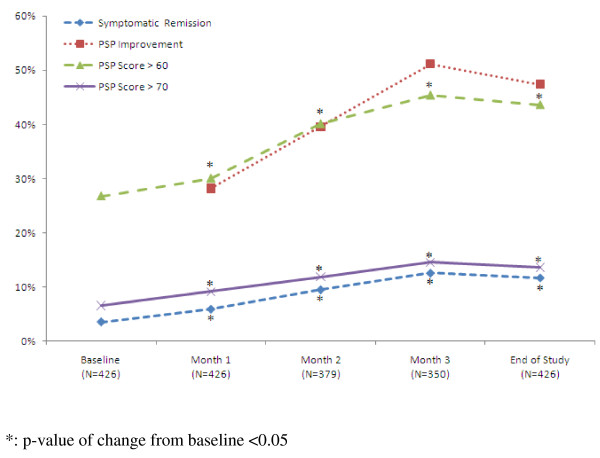
**Summary of Efficacy Result**. The symptomatic remission rate was 3.5% (95%CI, 1.98%, 5.74%) at baseline and improved to 11.7% (95%CI, 8.84%, 15.18%) at the end of study (p < 0.05). The criteria for PSP improvement was at least one 10-point interval on PSP scale. In the ITT population, subjects showed an increasing PSP improvement after treatment began. The improvement rate was increased from 28.1% (95%CI, 23.94%, 32.70%) at week 4 to 47.4% (95%CI, 42.59%, 52.28%) at the end of study.

PANSS and PAP total score both showed significant improvements after 12-week treatment (PANSS score, from 89.88 ± 29.20 to 72.72 ± 26.36; PSP score, from 47.07 ± 16.34 to 56.61 ± 14.32; both p < 0.05). The results of symptomatic remission are summarized in Figure 4. The symptomatic remission rate was 3.5% (95%CI, 1.98%, 5.74%) at baseline and improved to 11.7% (95%CI, 8.84%, 15.18%) at the end of study (p < 0.05). The criteria for PSP improvement was at least one 10-point interval on PSP scale. In the ITT population, subjects showed an increasing PSP improvement after treatment began. The improvement rate was increased from 28.1% (95%CI, 23.94%, 32.70%) at week 4 to 47.4% (95%CI, 42.59%, 52.28%) at the end of study.

AEs with occurrence ≥ 2% during the study are summarized in Table 7. There were 213 patients (50.0%) with ≥ 1 AE during study. The most commonly experienced AEs were disease progression (33 patients; 7.7%), upper respiratory tract infection (30 patients; 7.0%), extrapyramidal disorder (25 patients; 5.2%), insomnia (17 patients; 4.0%), and constipation (14 patients; 3.3%). Among the 30 schizophrenia events 27 were recorded as serious AEs.

**Table 7:** Adverse Events with Incidence ≧ 2%

Preferred Term	N = 426 N (%)
Patients with any Adverse Event	213 (50.0%)
Disease progression	33 (7.7%)*
Upper respiratory tract infection	30 (7.0%)
Extrapyramidal disorder	22 (5.2%)
Insomnia	17 (4.0%)
Constipation	14 (3.3%)
Anxiety	11 (2.6%)
Nasopharyngitis	11 (2.6%)
Diarrhoea	9 (2.1%)
Headache	9 (2.1%)
Somnolence	9 (2.1%)
Tachycardia	9 (2.1%)

*27 events were recorded as serious adverse events.

## Discussion

The severity of the symptoms and long-lasting, chronic pattern of schizophrenia can impact all areas of daily living including work or school, social contacts, and relationships. Treatment typically involves antipsychotic medications to stabilize the mood and treat the psychotic symptoms for individual patients. Paliperidone ER tablets have been approved in the USA and Europe for the treatment of schizophrenia based on three 6-week, placebo-controlled clinical trials in patients with acute symptoms of schizophrenia [13-15]. These studies indicate that paliperidone ER at dosages 3-15 mg/day was associated with statistically significant improvement (relative to placebo) in schizophrenia symptoms as measured by PANSS, personal and social functioning as measured by the PSP, and clinician's overall assessment as measured by CGI-S. Paliperidone ER was well tolerated in this patient population during acute treatment, with tolerability measured by low discontinuation rates and low adverse event burden [12,13,16]. The maintenance of social functioning is important treatment objective in the long-term management of schizophrenia. However, the aim of this study is to measure maintenance of social functioning with Personal and Social Performance scale (PSP) to assess treatment benefit in clinical trials. The 10-point PSP decrement is a clinically relevant measure of maintenance of functioning in patients stabilized with antipsychotic therapy. Paliperidone palmitate demonstrated a statistically significant treatment benefit in terms of maintenance of functioning [17].

The current phase IV, open-label, prospective study was conducted with the main objective of exploring the relationship between achieving symptomatic remission status by means of the 8 items of Positive and Negative Syndrome Scale (PANSS) and personal and social functioning by means of the Personal and Social Performance (PSP) scale in patients treated with flexibly dosed Paliperidone ER. The proportion of patients achieving the definition of symptomatic remission status was 3.52% with 95% C.I. [1.98%, 5.74%] at baseline and improved to 11.74% with 95% C.I. [8.84%, 15.18%] at the end of study of the ITT population.(Figure 4) The significant improvements in personal and social functioning that resulted subsequent to paliperidone ER treatment, as measured by the validated and reliable PSP instrument may be an important clinical consideration for patient treatment. Apart from improvement in positive and negative symptoms, medications that improve personal and social function may lead to better social integration and overall functioning [18,19]. The sensitivity demonstrated that the cut point 60 of PSP scale revealed best relationship between PSP scale and symptomatic remission. It would be useful to be able to assess the importance of both PSP scores and changes in PSP scores by relating them to real-life outcomes. Ultimately, a real-life assessment of PSP scores would have to be addressed by long-term observational studies incorporating relatively objective measures of social functioning, possibly drawing on multiple observers (e.g., clinicians, family members, friends, caregivers) as well as patient self-assessment [20-23]. The PSP may be a useful tool to assess social functioning and importantly to predict relapse, enabling management teams to intervene before the deleterious clinical and economic impact of relapse negatively affects the patient's course of illness. The high predictive value of the PSP criteria and relapse is particularly relevant in an illness such as schizophrenia where noncompliance and partial compliance to medication is substantial [24,25]. Patients with schizophrenia may present with negative, cognitive, disorganization and mood symptoms, which persist during periods of acute exacerbation when more overt positive symptoms are evident. The post-hoc analysis showed that acutely ill patients with or without predominant negative symptoms respond similarly to treatment with paliperidone ER [26].

The safety profile also demonstrated that paliperidone ER was well-tolerated without clinically significant changes after treatment administration. The most frequently reported adverse event was disease progression (33 patients, 7.7%), upper respiratory tract infection (30 patients, 7.0%), extrapyramidal disorder (25 patients, 5.2%), insomnia (17 patients, 4.0%) and constipation (14 patients, 3.3%). As well, one patient committed suicide, and another attempted suicide and was comatose in a vegetative state. Vital signs, such as weight, SBP, DBP, and pulse had no clinically significant change. Various clinical studies have demonstrated that paliperidone ER is safe and well-tolerated and have similar adverse event profile. Pooled safety data indicated that paliperidone ER was generally well tolerated. Discontinuations related to treatment-emergent AEs were similarly low for patients receiving paliperidone ER or placebo. Although the incidence of EPS-related AEs was higher in paliperidone ER-treated patients, primarily those receiving higher doses, the severity of EPS was very low throughout the study [27]. Therefore, no safety concerns were raised in this study [28]. In this study, short-term treatment with paliperidone ER significantly improved psychiatric symptoms and functioning, with no unexpected safety or tolerability findings. Paliperidone is the active metabolite of risperidone, and nearly half of the subjects were on risperidone prior to study entry. Oral risperidone may have failed to provide adequate efficacy in patients even though it is metabolized to paliperidone because of the short plasma half-life of paliperidone. This would make the case that paliperidone ER treatment would be more effective since it stays in blood circulation for an extended period of time and hence, the controlled drug release from the osmotic drug delivery system demonstrates clear formulation benefits as highlighted specifically in the title of this study. The symptomatic remission rate was 3.52% with 95% C.I. [1.98%, 5.74%] at baseline and improved to 11.74% with 95% C.I. [8.84%, 15.18%] at the end of study (p-value < 0.05). The results demonstrated an improvement in symptomatic remission rate after the 12-week treatment of paliperidone ER. Another study showed that the remission rate was increased from 43.9% at baseline to 51.7% at 12 weeks after aripiprazole treatment [29]. The original RSWG criteria requires 6 month duration, we have not used the criteria for remission as originally defined. There are three key limitations to the study. These are as follows. First, the study is the short study design. The study attempts to explore the relationship between symptomatic remission and function, however, this aspect of the investigation requires additional assessment for validity. A third limitation is the heterogeneous nature of the population, with some patients being remitted at baseline. Prospectively designed and longer-term studies are needed to further assess this finding.

## Conclusions

The diminished social functioning in schizophrenia is probably responsible for more burdens in patients, families, and care systems than residual symptoms. Finding a psychotropic treatment that improves social functioning is critically important. The clinical program of paliperidone ER was designed to incorporate the PSP as a measure of social functioning [30]. The result showed that the 11.74% patients with at least moderate severity of schizophrenia were evaluated as "mild" or better on PANSS scale by all 8 items after 12 weeks of treatment with paliperidone ER. There were also significant improvement in patients' functionality as measured by PSP improvement and score changes. The cut point 60 of PSP scale revealed best relationship between PSP scale and symptomatic remission. Besides, PANSS scale revealed better correlation with PSP scale rather than social functioning scale. Safety profile was also acceptable. This 12-week, multi-center, open-label; prospective study established the efficacy, safety, and tolerability of paliperidone ER and significantly showed that symptom severity and social functioning improve with paliperidone ER treatment. In the future, the correlations between PSP and PANSS to prove the close interplay between social functioning and psychopathology in the chronic course of schizophrenia should be further evaluated. The interaction of psychopathological states and psychosocial functioning determines the long-term course of schizophrenia and its treatment.

## Competing interests

This research was supported by Janssen-Cilag Taiwan, Johnson & Johnson. All the authors are clinical psychiatrists. The authors declare that they have no competing interests.

## Authors' contributions

YCY and MWH conceived the study, analyzed the data and prepared the manuscript. PPY participated in the study design and provided significant comments on the manuscript. PRT participated in the study design and helped to draft the manuscript. PWS, BJW, CHC, THL, ICL, WCC, CYL and KSC participated in the study design and helped to provide clinical service. All authors have read and approved the final version of the manuscript.

## Pre-publication history

The pre-publication history for this paper can be accessed here:

http://www.biomedcentral.com/1472-6904/12/1/prepub
